# Developmental mechanisms underlying improved contrast thresholds for discriminations of orientation signals embedded in noise

**DOI:** 10.3389/fpsyg.2014.00977

**Published:** 2014-09-08

**Authors:** Seong Taek Jeon, Daphne Maurer, Terri L. Lewis

**Affiliations:** ^1^Department of Vision Sciences, Institute for Applied Health Research, Glasgow Caledonian UniversityGlasgow, UK; ^2^Visual Development Laboratory, Department of Psychology, Neuroscience and Behaviour, McMaster UniversityHamilton, ON, Canada

**Keywords:** vision, contrast thresholds, internal noise, development, psychophysics

## Abstract

We combined an external noise paradigm with an efficient procedure for obtaining contrast thresholds (Lesmes et al., 2006) in order to model developmental changes in the effect of noise on contrast discrimination during childhood. Specifically, we measured the contrast thresholds of 5-, 7-, 9-year-olds and adults (*n* = 20/age) in a two alternative forced-choice orientation discrimination task over a wide range of external noise levels and at three levels of accuracy. Overall, as age increased, contrast thresholds decreased over the entire range of external noise levels tested. The decrease was greatest between 5 and 7 years of age. The reduction in threshold after age 5 was greater in the high than the low external noise region, a pattern implying greater tolerance of the irrelevant background noise as children became older. To model the mechanisms underlying these developmental changes in terms of internal noise components, we adapted the original perceptual template model (Lu and Dosher, 1998) and normalized the magnitude of performance changes against the performance of 5-year-olds. The resulting model provided an excellent fit (*r*^2^ = 0.985) to the contrast thresholds at multiple levels of accuracy (60, 75, and 90%) across a wide range of external noise levels. The improvements in contrast thresholds with age were best modeled by a combination of reductions in internal additive noise, reductions in internal multiplicative noise, and improvements in excluding external noise by template retuning. In line with the data, the improvement was greatest between 5 and 7 years of age, accompanied by a 39% reduction in additive noise, 71% reduction in multiplicative noise, and 45% improvement in external noise exclusion. The modeled improvements likely reflect developmental changes at the cortical level, rather than changes in front-end structural properties (Kiorpes et al., 2003).

## Introduction

Many aspects of basic vision improve rapidly over the first few years of life. For example, visual acuity, whether measured using visually-evoked potentials or preferential looking, improves rapidly between birth and 6 months of age and then continues to improve gradually until about 6 years of age (Norcia and Tyler, 1985; Chandna, 1991; Neu and Sireteanu, 1997). Front-end changes make a substantial contribution to the early improvements in basic visual abilities (e.g., Yuodelis and Hendrickson, 1986; Banks and Bennett, 1988). However, the development of cortical pathways appears to also contribute to the changes both during and after infancy (Banks and Bennett, 1988; Toga et al., 2006; Braddick and Atkinson, 2011).

The human brain is a complex system that consists of hundreds of anatomical structures and billions of neurons exchanging, at any given time, thousands of electrical and chemical signals through synapses connecting neurons in both nearby and remote parts of the brain. Like any other machinery, be it artificial or biological, neurons are not ideal transmitters of information. For example, identical signals from neighboring neurons do not elicit identical responses in the receiving neurons each time they are produced. This variability is observed even when external conditions, such as the sensory input or task goal, are kept as constant as possible (Cohn and Lasley, 1986; Faisal et al., 2008).

How is this fluctuation manifested at the level of visual behavior? As an illustration, a visual object may cause activation of neurons responsible for signaling its particular visual properties embedded in background activation of some neurons irrelevant to those properties. The spontaneous activation of these other neurons interferes with perceiving the visual signal clearly. The characteristic amount of this background variability added to the signal during processing is dubbed collectively as internal noise, which is known to provide an irreducible limit on detection. For example, the existence of an absolute contrast threshold that is higher than that of an ideal observer is evidence of such a limitation (Hecht et al., 1942; Rose, 1948; Barlow, 1956; Jones, 1959; Geisler, 2003).

Over the last several decades, visual psychophysicists have modeled the limitations inherent to the visual system in the contrast domain by measuring contrast thresholds for signals embedded in external noise (Burgess and Colborne, 1988; Pelli, 1990; Eckstein et al., 1997; Lu and Dosher, 1998; Solomon, 2002; Jeon et al., 2009; Klein and Levi, 2009). This method has been used to assay how the performance of visually normal adults is altered by changes in attention (Dosher and Lu, 2000; Lu et al., 2004) and by perceptual learning (Dosher and Lu, 1999; Gold et al., 1999; Dosher et al., 2004; Li et al., 2004). In addition, investigators have modeled changes occurring with development, both during childhood (Brown, 1994; Kiorpes and Movshon, 1998; Skoczenski and Norcia, 1998; Bogfjellmo et al., 2013; Falkenberg et al., 2014) and during aging (Pardhan et al., 1996; Betts et al., 2007), and the changes that occur after a history of early abnormal visual experience (Levi et al., 2007, 2008; Jeon et al., 2012; Falkenberg and Bex, 2014).

Typically, these approaches involve the manipulation of signals and experimentally controlled external noise in order to infer properties of the underlying perceptual process, which is presumably limited by the presence of various internal noise sources affecting perceptual sensitivity. By titrating the signal with the external noise, one can make inferences about how these internal noise sources affect sensory perception. When the relationship between the level of external noise and perceptual thresholds is measured and plotted in log-log coordinates, the resulting curve shows distinctive features, where the thresholds remain constant over the low external noise levels and then increase linearly as a function of external noise after a certain level of external noise. This curve is called the Threshold vs. Contrast or *TvC* curve. Systematic examination of the relative locations and shifts of *TvC* curves collected under different psychological manipulations or at different ages may reveal the underlying mechanisms responsible for changes in perceptual performance.

A few studies have used this approach to examine developmental changes in the levels of internal noise in the visual system of typically developing humans. Brown (1994) measured contrast detection and discrimination thresholds from infants whose age ranged from 49 to 51 days and adults using a minimum motion technique in which two gratings drifted in opposite directions. An observer used the participant's eye movements to determine the direction of the single grating (detection) or of the grating of higher contrast (discrimination). Threshold was defined as the minimum contrast difference for which the observer could make this determination accurately. The infants' thresholds were much more elevated for detection (factor of 50) than were their thresholds for discrimination (factor of 3). According to the modeling, the huge performance difference for detection between the infants and adults reflects higher intrinsic noise independent of stimulus contrast. Bogfjellmo et al. (2013) reached a similar conclusion when they measured sensitivity to the global direction of signal dots moving in a unitary direction against the noise dots moving in random directions.

Skoczenski and Norcia (1998) used visually evoked potentials (VEP) to record the electrophysiological responses of infants, aged 6–30 weeks, to sinusoidal gratings of varying contrast masked by varying amounts of temporally modulated noise. For each external noise level chosen for testing, the contrast of the grating was diminished into the background gradually until no VEP response was elicited. This contrast level was considered to be the contrast threshold for that noise level and was plotted, along with the contrast thresholds measured at other noise levels, to create a *TvC* curve from which the authors estimated the infants' internal noise. They found that the amount of internal noise in newborns was approximately nine times that of adults tested in the same way. They observed a rapid decrease in internal noise between 6 and 10 weeks of age, after which time the infants' internal noise was only 1.8 times greater than that of adults even though the contrast thresholds of infants were still higher by a much greater factor. Since overall contrast thresholds improved over the same time period during which internal noise decreased, the authors suggest that internal noise is a major limitation on infants' contrast sensitivity.

The one developmental study using macaque monkeys also reported decreases in internal noise with age. Kiorpes and Movshon (1998) trained young monkeys aged 1–18 months and adult monkeys to pull a bar or look in the direction of a grating presented on the left or right side of a monitor. The grating was presented either with or without noise frames temporally alternating with the stimulus frames. The authors used the method of constant stimuli to determine a signal contrast threshold for each noise contrast, and each individual's amount of internal noise was estimated from the resulting *TvC* curve. In accordance with the findings of Skoczenski and Norcia (1998), Kiorpes and Movshon (1998) observed a decrease in both internal noise and contrast thresholds with age. However, the decrease in contrast threshold could not be explained completely by the changes in intrinsic noise.

Until recently (Falkenberg et al., 2014), the findings on the development of human contrast detection/discrimination in noise have been restricted to early infancy: the ages tested have ranged only from 6 to 30 weeks of age (Brown, 1994; Skoczenski and Norcia, 1998). Falkenberg and colleagues used an equivalent noise paradigm to investigate and model the development and maturation of motion perception (detection, summation, and discrimination) in school-aged children (5–14 years) and adults. Measuring contrast thresholds at only two levels of external noise (no noise and high noise), they found a long developmental trajectory for only the discrimination of the motion direction, for which the contrast thresholds decreased continually into the adolescence. The authors modeled the decrease as arising from an improvement in sampling efficiency with no change in internal noise.

The previous studies with humans compared changes in performance across age groups by measuring a single *TvC* curve for each age group obtained at only one performance criterion (e.g., 75% correct). Although comparing single *TvC* curves provides valuable information about internal noise, measuring only one *TvC* curve cannot capture fully the mechanisms underlying performance change. In their detailed explanations of this point, Lu and Dosher (1998, 1999, 2008) and Lu et al. (2004) argued and demonstrated in a series of papers that more than one *TvC* curve must be measured at different performance criteria in order to characterize satisfactorily the mechanisms underlying various perceptual tasks. Measurement at multiple performance levels allows separate calculations of threshold ratios at each level of external noise. The additional data allow one to calculate separate estimates of internal additive noise, internal multiplicative noise, and template retuning (which is also called *excluding external noise*).

Measuring extra data points increases the power of the study, but also increases the time needed for testing, which can be especially problematic when testing children. To decrease the time required for data collection without sacrificing the quality of data, we used quick *TvC* (*qTvC*: Lesmes et al., 2006) to estimate contrast thresholds of four age groups (5-, 7-, 9-years-olds, and adults) at multiple performance levels across a wide range of embedded external noise levels. Specifically, we used *qTvC* to calculate contrast thresholds corresponding to 60, 75, and 90% correct performance levels for each of nine different external noise levels to obtain three *TvC* curves for each participant. With the aid of the *qTvC* method, we could collect data for each participant in less than 20 min. To model the mechanisms underlying developmental changes with age, we calculated an average *TvC* curve for each age group at each performance level. The current study is the first to evaluate the source of the known improvement in contrast thresholds with age in school-aged children (e.g., Ellemberg et al., 1999). To do so, we used the model of Lu and Dosher described above that has been successful in establishing the source of the limitations in adults tested at multiple levels of performance (Lu and Dosher, 1998, 1999, 2008; Lu et al., 2004).

## Materials and methods

### Participants

We tested four groups of participants: twenty 5.5-years-olds ± 3 months (mean age = 5.5 years, *SD* = 0.13, 5 female), twenty 7.5-year-olds ± 3 months (mean age = 7.5 years, *SD* = 0.11, 10 female), twenty 9.5-year-olds ± 3 months (mean age = 9.5 years, *SD* = 0.14, 11 female), and twenty adults ranging in age from 18.1 to 24.5 years (mean age = 19.6 years, *SD* = 1.45, 12 female). All participants in the final sample had passed a visual screening exam. Two additional 5.5-year-olds, one additional 7.5-year-old, and two additional adult participants were excluded because they did not pass the visual screening exam (see Section Procedure). The children were recruited from a database of children whose parents had volunteered to participate at the time of the child's birth. Children received a Junior Scientist certificate and a toy or a book voucher for their participation. Adults were volunteers or McMaster undergraduate psychology students who participated for course credit or $10 compensation.

### Apparatus and stimuli

The stimuli were presented on a 20 inch Sony Trinitron VGA color monitor with a pixel resolution of 640 × 480 and a 100 Hz refresh rate. The test stimuli were created in MATLAB (Mathworks, 2008). A stimulus sequence in a trial lasted 90 ms and consisted of nine alternating 10 ms patches of signal and noise in the following sequence: noise1-signal1-noise2-…-noise4-signal4-noise5. The signal was a Gaussian-windowed sinusoidal Gabor with a spatial frequency of 1 c/deg oriented ±45° from vertical. The alternation of the noise patches and the Gabor was fast enough that the noise appeared to be superimposed spatially on the Gabor. The luminance profile of the Gabor stimulus is described by the following equation:

(1) $$\begin{array}{l} L(x,y)=L_{0}(1.0+csin[2\pi f(xcos\theta +ysin\theta )]\\ \;exp[-(x^{2}+y^{2})/2\sigma^{2}] \end{array}$$

where *c* is signal contrast, σ is the standard deviation of the Gaussian window (1.86°), *f* is spatial frequency, and *L*_0_ is the background luminance which was set to the middle of the dynamic range of the display. The Gabor stimulus and noise patches were presented in a 7.8° × 7.8° frame when viewed from 57 cm. An external noise patch was composed of 0.1° × 0.1° pixel granules, the contrasts of which were sampled independently for each frame from a Gaussian distribution with a mean of 0 and one of the nine standard deviations (ranging from 2 to 33%, sampled in 3dB steps) as prescribed by the *qTvC*.

**Figure 1:**
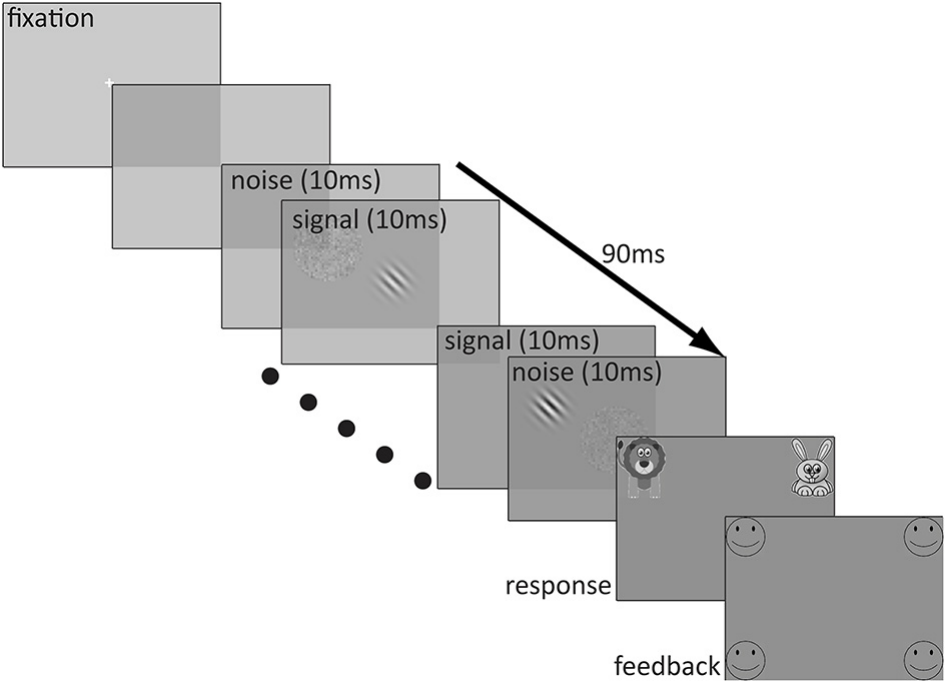
**Depiction of a sample trial sequence**.

Before each trial, a white fixation cross (0.6° × 0.6°, line width = 0.062°) was presented in the center of the monitor for 500 ms, followed by 250 ms of blank screen prior to the onset of the test stimulus. Immediately after the test stimulus, there appeared a response screen which consisted of an image of a cartoon lion in the upper left corner of the screen (bottom edge 3° above center and inner edge 8° to the left of center) and an image of a cartoon rabbit in the upper right corner of the screen (bottom edge 3° above center and inner edge 8° to the right of center). The size of each image was 12° × 12° and a white question mark was centered between them. This screen remained indefinitely until the participant made a response. Participants indicated whether the top of the Gabor was tilted to the right (rabbit) or to the left (lion) by pressing a key (the F key on the left side of the keyboard to indicate that the stimulus was angled to the left and the J key on the right side of the keyboard to indicate the stimulus was angled to the right). If participants preferred, they responded verbally by saying “left” or “lion” for a leftward choice and “right” or “rabbit” for a rightward choice to a blind experimenter who entered the response on a keyboard. If participants chose the correct answer, they received positive feedback in the form of four outlined, circular smiley faces (each 8° in diameter), one in each of the four corners of the monitor (closest edges 7° above and below center and inner edges 12° to the left and right of center) and an encouraging cheering sound. However, if participants responded incorrectly, they received four outlined frowning faces of the same size and location and heard a “D'oh!” sound indicating that their choice was incorrect. A typical trial sequence is depicted in Figure 1.

### Procedure

Prior to any procedures, we obtained informed consent from participants or their parents. We also obtained assent from the children 8 years and older. Adult participants and parents of children were provided with a debriefing form upon completion of the experiment. Our experimental procedures were cleared by the McMaster Research Ethics Board.

#### Visual screening procedure

All participants in the final sample had normal or corrected-to-normal vision, for their age. The visual screening exam included tests of linear letter acuity, binocular fusion, and stereo acuity. Adults, 9- and 7-year-olds were required to have a linear letter acuity enabling them to read correctly all but two letters on the 20/20 line in each eye when tested monocularly with the Lighthouse Distance Visual Acuity Test chart. The 5-year-olds had a linear letter acuity of at least 20/25 when tested with the Goodlite Crowding cards. If necessary, participants were given spectacle corrections of up to −1.5 dioptres to ensure that any myopic error was too small to interfere with vision at the testing distance of 57 cm. Participants were required to have worse acuity with an added +3.00 dioptre lens to rule out hypermetropia (farsightedness) of more than 3 dioptres. Binocular fusion was assessed using the Worth 4-Dot Test, and stereoacuity was assessed with the Titmus Fly Stereotest. Participants were required to show evidence of binocular fusion and stereoacuity of at least 100 arcsec for the 5-year-olds and 40 arcsec for the older participants.

#### Experimental procedure

Participants sat in a darkened room and viewed the stimuli binocularly. The experimental procedure consisted of a demonstration, a criterion, a practice run, and a test run.

***Demonstration***. Participants were told that the pattern they were going to see “looks similar to a Ruffles® potato chip.” The experimenter showed participants a vertical Gabor with no noise and told them “the lines on the chip make it look like it is standing straight up and down.” This screen was presented until participants agreed verbally that the Gabor was oriented vertically. The experimenter then told participants that “the Ruffles® potato chip in the computer game will be tilted and the goal was to decide whether the top of the Ruffles® potato chip was tilted toward the right (toward the rabbit) or toward the left (toward the lion).” The experimenter then showed participants a static right-tilted Gabor in the center of a screen containing the previously described cartoon rabbit and lion and explained, “This is what the chip will look like when it is tilted toward the right (toward the rabbit).” They were shown this screen until they agreed verbally that the Gabor was now tilted toward the rabbit. They were then shown the vertical Gabor again, followed by a static left-tilted Gabor on a screen containing the cartoon animals and were told that “This is what the chip will look like when it is tilted toward the left (toward the lion).” This screen was presented until participants agreed verbally that the Gabor was now tilted toward the lion. The participants were then asked to indicate verbally which way two practice Gabors were tilted. As in the first two trials, the two practice Gabors were presented without noise but were shown for only 1 s each. All participants responded correctly to these two 1-s practice trials and were given feedback by the computer program and by the experimenter.

***Criterion***. Next, we ensured that participants understood the task by testing them with a criterion session in which they were shown static Gabors at 50% contrast in no noise for 90 ms and were required to give four consecutive correct answers. The participants received feedback from the computer program for each of their responses. The experimenter then explained that sometimes the “Ruffles® potato chip will be sprinkled with salt and pepper and will look fuzzy.” On the computer screen, we presented one right-tilted Gabor alternated with a 50% contrast noise patch and then a similar left-tilted Gabor with 50% noise and told participants which way they were tilted. Both Gabors were presented until the participant indicated that they saw which way the “Ruffles® potato chip” was tilted beneath the salt and pepper. They were then tested with a second criterion session in which the Gabors were presented for 90 ms with 50% noise. Again, they were required to give four consecutive correct answers. Participants were required to pass each of the criterion sessions in no more than three blocks of four trials, and all did so in the first or second block.

***Practice***. After participants passed both criterion sessions successfully, the experimenter presented a 24-trial practice run. The stimuli used in the 24-trial practice run were generated by the *qTvC* program and were identical to the first 24 trials used in the test run. The computer program generated the three parameters of the resulting *TvC* curve based on the 24 practice trials: the critical noise (*N_c_*), the optimal contrast threshold (*C*_0_), and the common slope of the psychometric function (η) and these parameters were recorded by the experimenter.

***Test run***. The test run was identical to the practice run except that it consisted of 240 trials differing in stimulus contrast and noise levels, as generated by the *qTvC* paradigm. The stimulus space for the *qTvC* procedure included nine possible external noise contrasts ranging from 2 to 33% (in 3dB steps), and signal contrast levels sampled from a pool of 40 possible contrast levels ranging from 0 to 90% (in 1dB steps). Participants who requested a break were given a 5-min quiet break in the testing room. At the end of the test run, the program reported thresholds corresponding to three levels of accuracy: 60, 75, and 90%. Each experimental session lasted approximately 40 min plus approximately 5 min for visual screening.

### Modeling

To quantify and model the improvement with age, we adopted the original Perceptual Template Model (*PTM*; Lu and Dosher, 1998), developed previously to characterize changes in perceptual performance with attention (Lu and Dosher, 1998; Dosher and Lu, 2000) and perceptual learning (Dosher and Lu, 1999; Lu et al., 2004). A detailed description of the *PTM* can be found in one of the cited papers. Briefly, overall performance of an observer, expressed in *d*′, is limited by the following three noise sources in the *PTM*: (1) external noise (*N_ext_*), the strength of which is known to the experimenter, (2) internal additive noise (*N_add_*), an irreducible amount of variability inside a system determining the lower bound on performance (Barlow, 1956; Pelli, 1990), and (3) multiplicative noise (*N_mul_*), an independent noise source, the strength of which is proportional to the stimulus strength (Green and Swets, 1974; Legge and Foley, 1980). The initial signal and noise composite may be subject to a non-linearity (γ) in the system (Nachmias and Sansbury, 1974; Kontsevich et al., 2002). Combined, the overall performance of a system is fundamentally determined by the signal-to-noise ratio:

(2) $$\begin{array}{l} d^{\prime }=\frac{S}{N_{total\;noise\;sources}}\\ \;=\frac{{\left(\beta c\right)}^{\gamma }}{\sqrt{N_{ext}^{2\gamma }+N_{mul}^{2}[{\left(\beta c\right)}^{2\gamma }+N_{ext}^{2\gamma }]+N_{add}^{2}}} \end{array}$$

where *c* is the contrast of the signal and β represents the gain or amplification factor on the signal after a perceptual template which is tuned to the relevant dimension of stimulation (e.g., contrast in the current case). Rearranging the equation for the contrast threshold yields,

(3) $$c_{\tau }=\frac{1}{\beta }{\left[\frac{\left(1+N_{mul}^{2}\right)N_{ext}^{2\gamma }+N_{add}^{2}}{\left(\frac{1}{{d^{\prime }}^{2}}-N_{mul}^{2}\right)}\right]}^{\frac{1}{2\gamma }}$$

**Figure 2:**
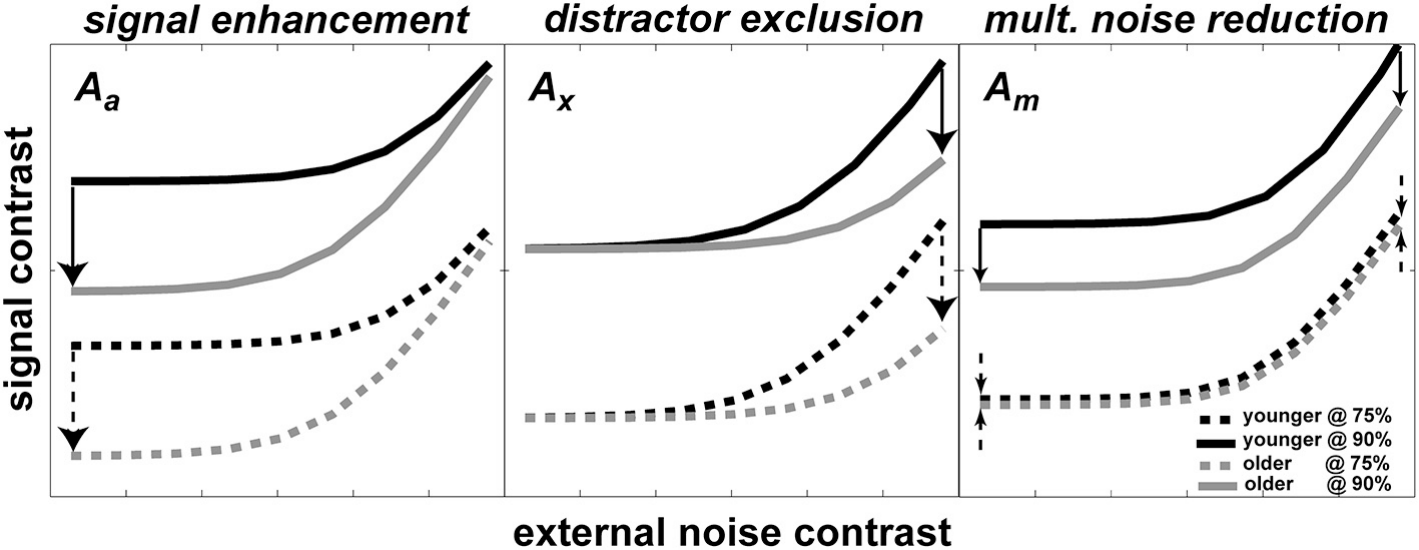
**Possible mechanisms of development predicted by *PTM***. Each panel shows how *TvC* curves at two performance criteria would change during development for changes in one of three *PTM* parameters where *A_a_* represents internal additive noise (left panel), *A_x_* represents distractors exclusion (middle panel), and *A_m_* represents multiplicative noise (right panel). In all three panels, darker lines represent younger age groups whereas lighter lines represent older groups. Solid lines represent more stringent performance criteria; dotted lines represent less stringent performance criteria. Arrows represent the hypothetical size and direction of change in performance during development.

According to the *PTM*, improvement in performance through development can be modeled by changes in one or more of the noise sources. Each panel in Figure 2 shows a hypothetical pattern of performance change when only one of the three mechanisms mentioned above is in operation: (1) *stimulus enhancement* (left panel)—represents improvement caused by a reduction in internal additive noise. In this case, the improvement will be shown in the low external noise region. (2) *External noise exclusion* (middle panel)—represents the ability to suppress or filter out irrelevant information (i.e., external noise). As opposed to case (1), this pattern of improvement will be shown when external noise is high. (3) *Internal multiplicative noise reduction* (right panel)—a reduction of internal multiplicative noise will improve performance over the entire range of external noise levels.

Assuming all three mechanisms are at work, we can rewrite the above equation to accommodate the developmental changes in our current data as following:

(4) $$c_{\tau }=\frac{1}{\beta }{\left[\frac{\left(1+{\left(A_{m}^{\left(i\right)}N_{mul}\right)}^{2}\right){\left(A_{x}^{\left(i\right)}N_{ext}\right)}^{2\gamma }+{\left(A_{a}^{\left(i\right)}N_{add}\right)}^{2}}{\left(\frac{1}{d^{\prime }{}^{2}}-{\left(A_{m}^{\left(i\right)}N_{mul}\right)}^{2}\right)}\right]}^{\frac{1}{2\gamma }}$$

where the index (*i*) denotes the age group. To quantify the relative contributions from each or combinations of the noise sources to the improvements with age, three extra coefficients *A*s with subscripts corresponding to each noise source are used. In this form, the relative improvements with age are quantified against the performance of 5-year-olds, where we set *A^5yro^_a_* = *A^5yro^_x_* = *A^5yro^_m_* = 1.

Figure 2 also illustrates the important property of the *PTM* for contrast thresholds predicted at two different performance criteria for each mechanism. In each panel, there are two pairs of darker and lighter lines representing hypothetical *TvC* curves for younger and older observers, respectively. Each pair of curves was drawn at two different performance criteria (e.g., solid lines represent 90% correct performance level and broken lines represent 75% correct performance level). The direction of the arrows represents the direction of improvement and the size of the arrows approximately matches the magnitude of improvement. Inspection of the figure highlights how the magnitude of change can be contingent upon performance criteria. In the cases of signal enhancement (left panel) and distractor exclusion (middle panel), for example, the size of improvement is constant regardless of the criteria. On the other hand, the size of improvement increases as the performance criterion becomes more stringent in the case of multiplicative noise reduction (right panel). Therefore, measuring multiple *TvC* provides strong constraints and is useful in distinguishing between mixtures of mechanism in the hierarchical model testing in the *PTM*.

## Results

**Figure 3:**
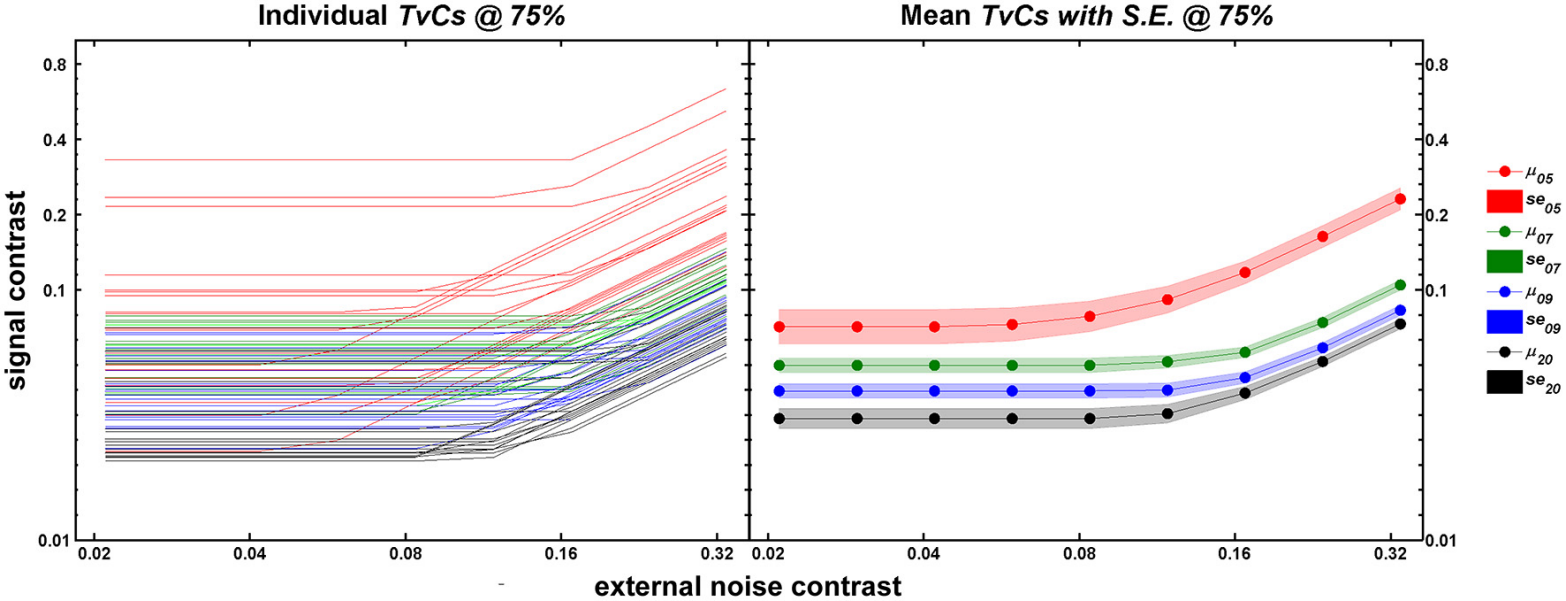
**Individual (left panel) and mean (right panel) *TvC* curves at 75% correct performance**. In both panels, abscissa represents external noise strength and ordinate represents contrast thresholds obtained after 240 trials of *qTvC*. Age groups are color-coded as red, green, blue, and black for 5-, 7-, 9-year-olds, and adults, respectively. The left panel represents individual *TvC* curves (*n* = 20/age group) at the 75% correct performance level for 240 trials of *qTvC*. The right panel represents averaged *TvC* curves for each age group. The shaded areas represent ±1 *SE*.

Figure 3 shows the *TvC* curves at 75% correct performance for each age group (5-, 7-, 9-year-olds, and adults in red, green, blue, and black respectively). The left panel shows the individual outputs (*n* = 20/group) after running 240 trials of *qTvC*. The right panel shows the mean for each age group with the shaded regions representing ±1 s.e.m. at each noise level. As age increases, the average performance improves (shown as decreases in contrast thresholds) over the entire noise range tested. The improvement seems greatest, especially in the high noise region, between 5 and 7 years of age, after which the improvement with age becomes more gradual. This pattern is evident, even when one takes into account the greater variability in performance at age 5.

**Figure 4:**
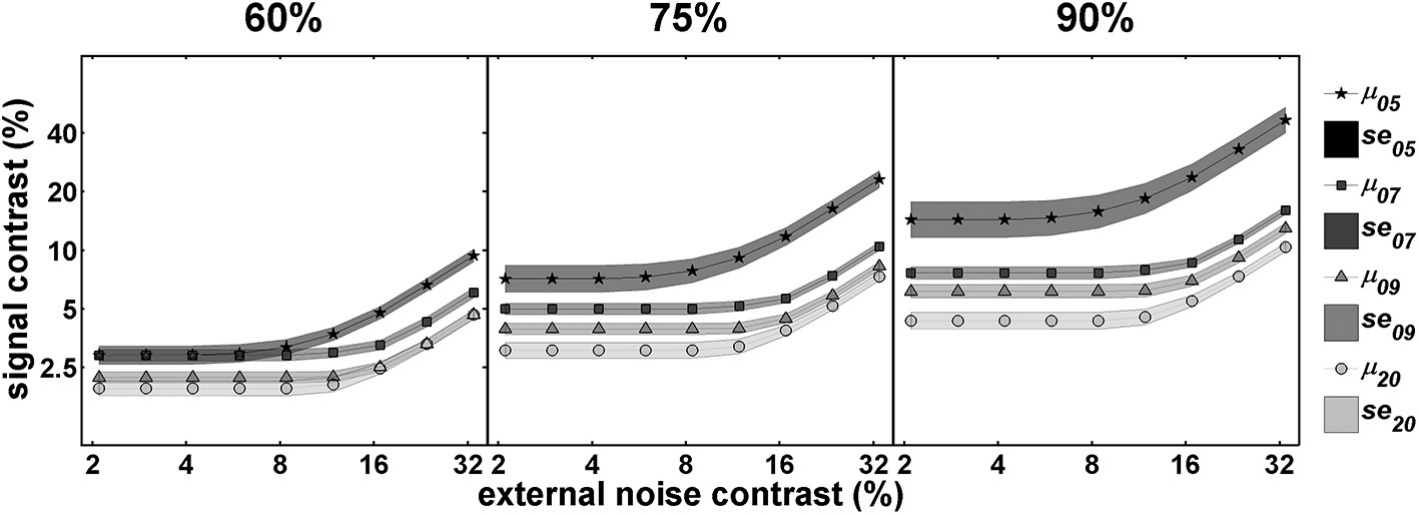
**Mean *TvC* curves at three performance criteria**. This figure illustrates how the magnitude of the change in performance with age, depicted by relative shifts among *TvC* curves, can be dependent upon performance criteria. Illustrations are for performance criteria of 60% **(left panel)**, 75% **(middle panel)**, and 90% **(right panel)**. In all panels darker lines represent younger age groups whereas lighter lines represent older groups. See text for more details.

Figure 4 shows the developmental data over three different performance levels. As mentioned in the Introduction and the Section Modeling above, multiple *TvCs* at different criteria provides stronger constraints in distinguishing the mixtures of mechanisms (Dosher and Lu, 1999; Klein and Levi, 2009). Qualitatively, there is an increase in the threshold ratios among different age groups across the entire external noise range as a more stringent performance criterion is implemented, implying the impact of multiplicative noise on the change in contrast thresholds. From the disproportionate changes in threshold ratios between the low and high external noise region across different performance criteria, we can also infer that signal enhancement and external noise exclusion may be at work at the same time.

**Figure 5:**
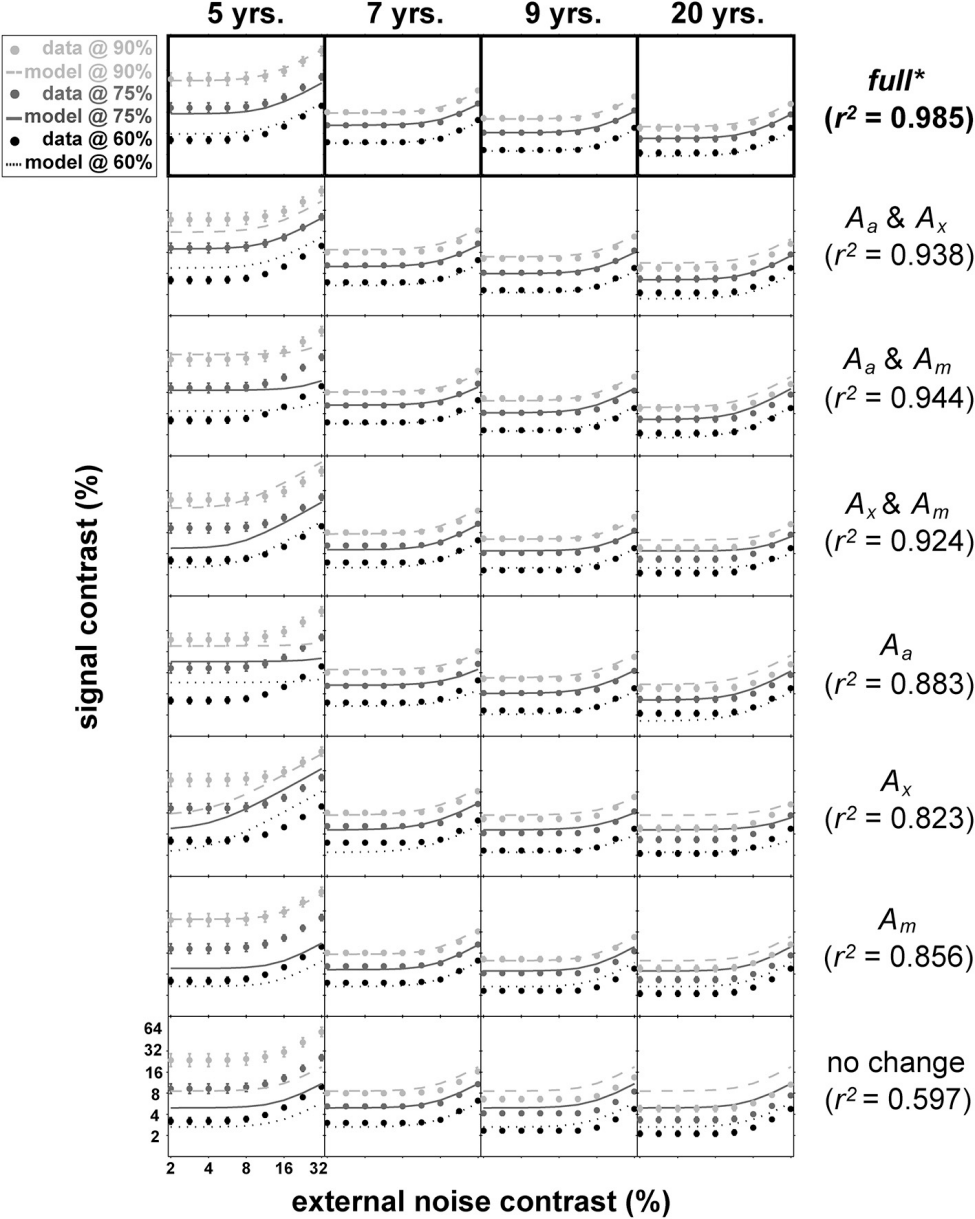
**Results of nested modeling**. This figure matrix shows the results of nested *PTM* modeling arranged by age (columns) and model layers (rows). In each panel, dots represent mean contrast thresholds at each noise level tested using *qTvC*, and smooth curves represent model fit to the data. The performance criteria become less stringent as the colors become darker. Next to the figure matrix, goodness-of-fit statistics for a given model layer are provided. Statistical comparisons among model layers reveal that the “full” model in the top row is the best model, highlighted by an asterisk and thick black outlines around the top panels.

Figure 5 shows the mean data for each age group and the result of nested model fitting of these mean data using the equation (4). In this figure, age group is arranged column-wise while different models used for fitting are arranged in rows. Each panel contains data (shown as dots) at three performance levels (60, 75, and 90% correct) with error bars and the resulting model fits shown as lines. The age-related improvements can be seen across the columns as a gradual decrease in thresholds regardless of the performance levels. Note that the distance between the contrast thresholds at different performance levels is distinctively wider in 5-year-olds' data than the data from the remaining age groups.

The total number of data points used in this fitting procedure was 108 (9 noise levels × 3 performance criteria × 4 age groups). There are four layers of models with each layer of the same model containing the same number of parameters. For example, the most saturated layer has a model with 13 parameters (denoted as “full” in Figure 5) whereas the most parsimonious layer has a model with only four parameters (“no change” in Figure 5). There are a total of eight possible models across layers. With each model, we calculated goodness-of-fit (*r*^2^) (Equation 5) and compared them statistically (Equation 6) between layers.

(5) $$r^{2}=1-\frac{\sum {\left[\log\left(c_{\tau }^{PTM}\right)-\log(c_{\tau }^{data})\right]}^{2}}{\sum {\left[\log\left(c_{\tau }^{data}\right)-mean(\log\left(c_{\tau }^{data}\right))\right]}^{2}}$$

Of all 22 comparisons, no models in the sub-layers produced statistically equivalent goodness-of-fit compared to the goodness-of-fit for the most saturated model in the top layer with 13 parameters (top row in Figure 5, highlighted with boldface).

(6) $$F\left(df_{1},df_{2}\right)=\frac{(r_{upper}^{2}-r_{lower}^{2})/df_{1}}{(1-r_{upper}^{2})/df_{2}}$$

where *df*_1_ = *k_upper_* − *k_lower_*, and *df*_2_ = *N − k_upper_*. The *k*s are the number of parameters in each model to fit the data, and *N* is the number of data points to fit. We calculated bootstrapped confidence intervals for the best fitting *PTM* parameters by fitting the model 1000 times to the synthetic *TvC* thresholds resampled from each of three *qTvC* parameter distributions obtained from our observers. The pair of number in parentheses represents the confidence interval for each parameter. The full list of model parameters can be found in Appendix A in Supplementary material and the complete results of nested model comparisons are provided in Appendix B in Supplementary material.

The best model (top row in Figure 5) provided an excellent fit (*r*^2^ = 0.985) to the contrast thresholds at multiple levels of performance (60, 75, and 90%) across a wide range of external noise levels. The model suggests that a mixture of mechanisms underlies the developmental changes: the improvements in contrast thresholds over ages were best modeled by a combination of reductions in internal additive and multiplicative noise and improvements in excluding external noise (see Table 1). In line with the data, the improvement was greatest between 5 and 7 years of age, accompanied by a 38.6% reduction in additive noise, 70.7% reduction in multiplicative noise, and 45.1% improvement in external noise exclusion.

**Table 1:**
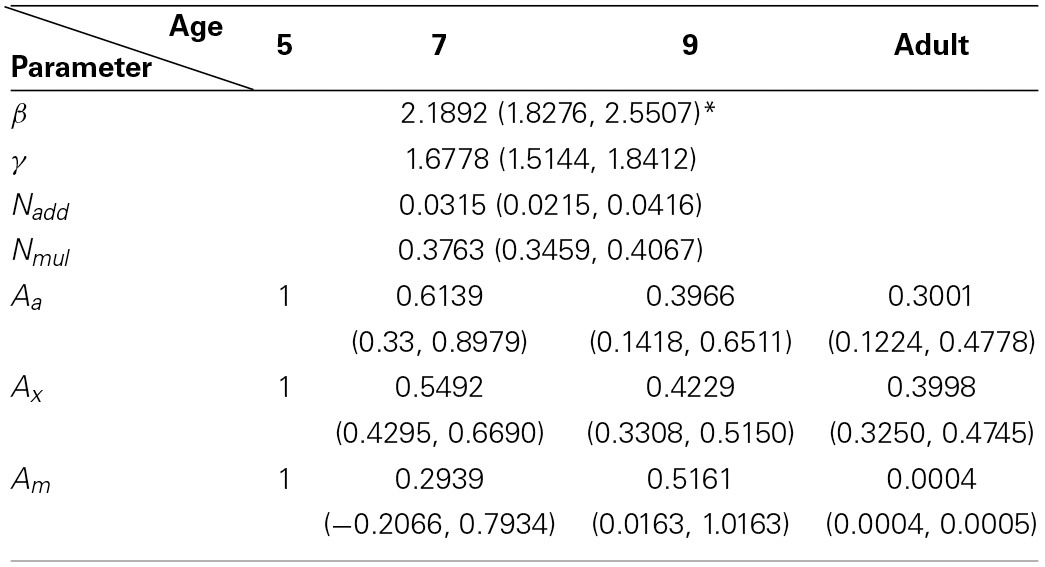
***PTM* parameter outputs from the best model**.

*β, gain from template matching*.

*γ, non-linearity exponent*.

*N_add_, standard deviation of additive noise*.

*N_mul_, standard deviation of multiplicative noise*.

*A_a_, a developmental parameter associated with signal enhancement*.

*A_x_, a developmental parameter associated with external noise exclusion*.

*A_m_, a developmental parameter associated with internal multiplicative noise reduction*.

^*^The numbers in parentheses represent bootstrapped confidence intervals for the best PTM parameters by fitting the synthetic thresholds resampled 1000 times from the three qTvC parameter distributions obtained from our observers.

**Figure 6:**
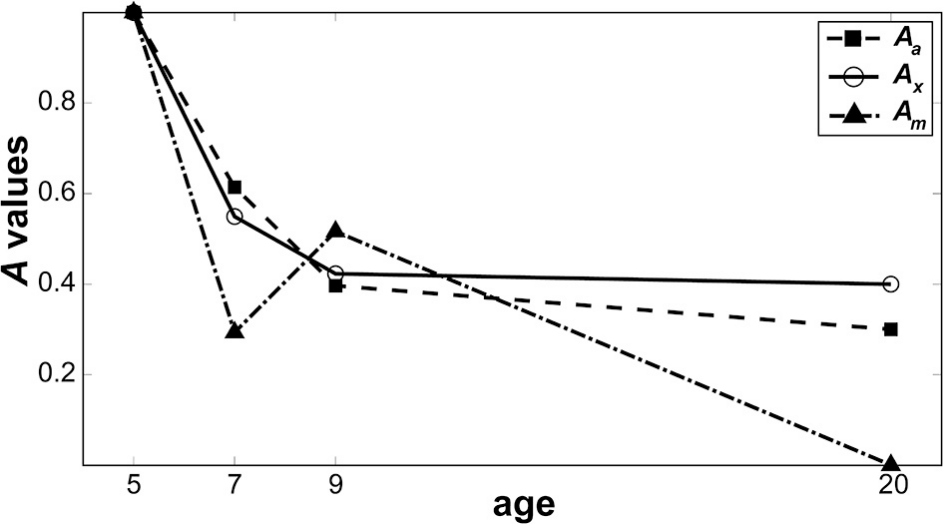
**Change in noise components as a function of age**. Parameters from the best model are plotted to show how the level of each noise component changes as a function of age when normalized against 5-year-olds. Filled squares represent internal additive noise, open circles represent the ability to exclude distractors, and filled triangles represent multiplicative noise.

Figure 6 shows relative changes in each noise source with age. While both internal additive noise (*A_a_*) and the ability to exclude distractors (*A_x_*) seem to reach adult levels at the age of 9, multiplicative noise continues (*A_m_*) to decrease after age 9 (the oldest age of child tested here).

## Discussion

The purpose of the current study was to measure contrast thresholds embedded in a wide range of external noise in four age groups and to model the developmental improvements in contrast thresholds in terms of changes in limiting factors affecting visual performance. With a *qTvC* procedure (Lesmes et al., 2006), contrast thresholds at multiple performance criteria across nine external noise levels were estimated quickly in children and adults. We modeled our data with *PTM* to investigate whether the developmental improvement in contrast threshold with age can be modeled by a combination of reduction in internal additive and multiplicative noise components as well as the improvement in filtering out irrelevant information.

In a previous study (Jeon et al., 2012), we included a task similar to the current experiment as one of the outcome measures to gauge the effect of video game training on the vision of adult congenital cataract patients and normal adult controls. In doing so, we applied the *qTvC* for the first time to collect 240 trials of data before and after the video game training. In the current study, we were able to collect data on 80 observers from a broad age range, highlighting the efficiency of *qTvC* in measuring and specifying the performance space defined across a wide range of noise and signal intensity.

Previous developmental studies consistently reported that infants and children are worse than adults at detecting or discriminating signals embedded in noise (Brown, 1994; Skoczenski and Norcia, 1998; Falkenberg et al., 2014). Those studies with infants found that the immaturity could be explained by higher internal additive noise. On the other hand, Falkenberg et al. (2014) found that poor sampling efficiency is responsible for the immaturity in motion discrimination of children and adolescents while the internal noise played no role in the development of motion discrimination after age 5, the youngest age tested. Bogfjellmo et al. (2013) reached a similar conclusion about sensitivity to the global direction of signal dots moving in a unitary direction against noise dots moving in random directions. Our work contrasts with these previous studies because we used a method that allowed us to distinguish between additive and multiplicative internal noise. At least for our task (contrast thresholds for orientation discrimination), internal additive noise was higher than in adults as late as age 7 and internal multiplicative noise was higher even at age 9, the oldest group of children tested. Specifically, the model identified three limits on 5-year-olds' contrast thresholds: (1) internal additive noise, (2) internal multiplicative noise, and (3) insufficient filtering of external noise.

First, our model showed that internal additive noise decreases with age for measurements of orientation discrimination in the contrast domain. Compared to 5-year-olds, there is a 39% reduction in 7-year-olds, a 60% reduction in 9-year-olds, and a 70% reduction in adults. These reductions account for the improvements in performance in the low external noise region.

Second, our data showed that the age-related change in contrast thresholds is dependent upon performance criteria, which is indicative of change in the level of internal multiplicative noise. As illustrated in Figure 4, the performance difference among age groups increased when higher accuracy was required. According to our modeling results, internal multiplicative noise also decreases with age. Compared to 5-year-olds, there is a 48–71% reduction by age 7–9 of internal multiplicative noise, and a complete elimination of it in adults, corresponding to a reduction of nearly 100%. There are competing points of view on what is responsible for the rising thresholds with increasing noise, masking, or pedestal values: multiplicative noise vs. contrast gain control. Empirically the influence of multiplicative noise is indistinguishable from that of a contrast-gain control mechanism (Dao et al., 2006; Klein and Levi, 2009; Chen et al., 2014). In a developmental study of contrast gain control using VEP (Garcia-Quispe et al., 2009), human infants from 15 to 28 weeks showed little contrast gain control compared to the older observers. This is the first study to make measurements of this factor in older children. The continuous reduction of multiplicative noise throughout childhood shown in our study might suggest a long developmental trajectory in the contrast gain control mechanism. Alternatively, or in addition, it might reflect a long developmental trajectory for the reduction in multiplicative noise.

A third factor responsible for the age-related improvements we observed in contrast threshold was an improvement in the ability to filter out external noise, which is reflected as improvements in contrast thresholds at high external noise levels. Compared to 5-year-olds, the impact of the external noise on discrimination was reduced by 45% in 7-year-olds, 58% in 9-year-olds, and 60% in adults. Studies of perceptual learning (Lu and Dosher, 1999; Chung et al., 2005), and aging (Betts et al., 2007) confirm that performance can be improved by increased exclusion of external noise, achieved by retuning an internal template to the stimulus property relevant to a given task so that it filters out incoming noise.

During development, channel reweighting (e.g., Lu and Dosher, 1999) of the sensory inputs likely becomes increasingly selective and tuned to the most relevant channel for forming perceptual decisions for a given task. Thus, given the shallow slope of the psychometric function in 5-year-olds, their response might be more similar than that at older ages across a wider range of input signals, the strength of which varies with external noise. This, in turn, would lower the differential signal-to-noise ratios around the relevant channels. For the visual system of 5-year-olds, this insensitivity to contrast might make it difficult to choose selectively the optimal channel for discrimination. In fact, substantial evidence indicates that young children are not optimal in selecting and processing the visual information that is most relevant to a given task. For example, the literature on visual selective attention indicates that children are not as good as adults at filtering out irrelevant background stimuli (Enns and Girgus, 1985; Ridderinkhof and Van Der Molen, 1995; Goldberg et al., 2001), with children as old as 10 years being affected more by distractors than adults (Goldberg et al., 2001). As reported by our best model output (Figure 6), it seems that the ability to cull external noise improves continually until 9 years of age.

Even though physiological changes such as pruning of excessive synaptic connections within the primary visual cortex, still occur until early adolescence (Huttenlocher et al., 1982; Garey and De Courten, 1983), it is unclear how much front-end changes in the structure or morphology of the early visual pathway can account for the developmental changes observed in our current age groups. In their study evaluating the developmental changes in contrast threshold and intrinsic noise using infant monkeys, Kiorpes and Movshon (1998) argued that changes in both additive and non-additive sources of noise contribute to the fall of the contrast thresholds during development. To arrive at this conclusion, they considered additive noise to represent the limiting factors in the early visual pathways and non-additive noise to represent “central” limiting factors, which might be tantamount to our internal multiplicative noise reduction and distractor exclusion. The documented changes in the striate visual pathway that continue well into adolescence may be responsible for such changes (Shaw et al., 2008; Pinto et al., 2010).

Even though the length of our procedure was reduced with the aid of *qTvC*, it might still be possible that children are simply less motivated or have a poorer understanding of the task. However, it is unlikely that worse performance in younger age groups was caused by a lack of motivation or understanding. First, we made sure that children understood the task by showing them demonstration trials, documenting their understanding with criterion trials, and familiarizing them with the test by having them complete a full session of *qTvC* before the data to be used were collected. Second, we kept the children motivated throughout the task by adding humorous auditory feedback when the child answered correctly. Although they were told that they could stop at any time, no child decided to discontinue the study, and all children seemed to enjoy the experimental procedure. Third, the *qTvC* algorithm kept performance much higher than chance level on most trials. Therefore, our observed effects were most likely a consequence of factors related to visual sensitivity and minimally affected by cognitive immaturity or lack of motivation.

In summary, the results from the current study suggest that the contrast sensitivity of 5-year-olds is limited by higher levels of internal additive and multiplicative noise and higher susceptibility to irrelevant background information. There are rapid decreases in these limitations until age 7 and gradual reductions thereafter, with the reduction in multiplicative noise continuing past age 9, the oldest age tested here. It can be hypothesized that these limitations at age 5 can explain previous observations of poorer thresholds and decreased psychometric slopes compared to older ages. Our model using a mixture of reductions in internal additive noise, reductions in internal multiplicative noise, and an improvement in the ability to filter out external noise can account well for the age-related improvements in contrast threshold.

### Conflict of interest statement

The authors declare that the research was conducted in the absence of any commercial or financial relationships that could be construed as a potential conflict of interest.

## References

[B1] Banks M. S. Bennett P. J. (1988). Optical and photoreceptor immaturities limit the spatial and chromatic vision of human neonates. J. Opt. Soc. Am. A 5, 2059–2079 10.1364/JOSAA.5.002059 3068345

[B2] Barlow H. B. (1956). Retinal noise and absolute threshold. J. Opt. Soc. Am. A 46, 634–639 13346424 10.1364/josa.46.000634

[B3] Betts L. R. Sekuler A. B. Bennett P. J. (2007). The effects of aging on orientation discrimination. Vision Res. 47, 1769–1780 10.1016/j.visres.2007.02.016 17466355

[B3a] Bogfjellmo L-G. Bex P. J. Falkenberg H. K. (2013). Reduction in direction discrimination with age and slow speed is due to both increased internal noise and reduced sampling efficiency. Invest. Ophthalmol. Vis. Sci. 54, 5204–5210 10.1167/iovs.13-12005 23800764 PMC3736759

[B4] Braddick O. Atkinson J. (2011). Development of human visual functions. Vision Res. 51, 1588–1609 10.1016/j.visres.2011.02.018 21356229

[B5] Brown A. M. (1994). Intrinsic contrast noise and infant visual contrast discrimination. Vision Res. 34, 1947–1964 10.1016/0042-6989(94)90025-6 7941396

[B6] Burgess A. E. Colborne B. (1988). Visual signal detection. IV. Observer inconsistency. J. Opt. Soc. Am. A. 5, 617 3404312 10.1364/josaa.5.000617

[B7] Chandna A. (1991). Natural history of the development of visual acuity in infants. Eye (Lond). 5(Pt 1), 20–26 10.1038/eye.1991.4 2060665

[B7a] Chen G. Hou F. Yan F-F. Zhang P. Xi J. Zhou Y. (2014). Noise provides new insights on contrast sensitivity function. PLoS ONE 9:e90579 10.1371/journal.pone.0090579 24626135 PMC3953123

[B8] Chung S. T. Levi D. M. Tjan B. S. (2005). Learning letter identification in peripheral vision. Vision Res. 45, 1399–1412 10.1016/j.visres.2004.11.021 15743610 PMC2741315

[B9] Cohn T. E. Lasley D. J. (1986). Visual sensitivity. Annu. Rev. Psychol. 37, 495–521 10.1146/annurev.ps.37.020186.002431 3963783

[B10] Dao D. Lu Z.-L. Dosher B. A. (2006). Adaptiation to sine-wave gratings selevtively reduces the contrast gain of the adapted stimuli. J. Vis. 6, 739–759 10.1167/6.7.6 16895456

[B11] Dosher B. A. Liu S.-H. Blair N. Lu Z.-L. (2004). The spatial window of the perceptual template and endogenous attention. Vision Res. 44, 1257–1271 10.1016/j.visres.2004.01.011 15066390

[B12] Dosher B. A. Lu Z. L. (1999). Mechanisms of perceptual learning. Vision Res. 39, 3197–3221 10.1016/S0042-6989(99)00059-0 10615491

[B13] Dosher B. A. Lu Z. L. (2000). Mechanisms of perceptual attention in precuing of location. Vision Res. 40, 1269–1292 10.1016/S0042-6989(00)00019-5 10788639

[B14] Eckstein M. P. Ahumada A. J. Watson A. B. (1997). Visual signal detection in structured backgrounds. II. Effects of contrast gain control, background variations, and white noise. J. Opt. Soc. Am. A 14, 2406–2419 9291610 10.1364/josaa.14.002406

[B15] Ellemberg D. Lewis T. L. Liu C. H. Maurer D. (1999). Development of spatial and temporal vision during childhood. Vision Res. 39, 2325–2333 10.1016/S0042-6989(98)00280-6 10367054

[B16] Enns J. T. Girgus J. S. (1985). Developmental changes in selective and integrative visual attention. J. Exp. Child Psychol. 40, 319–337 10.1016/0022-0965(85)90093-1 4045383

[B17] Faisal A. A. Selen L. P. Wolpert D. M. (2008). Noise in the nervous system. Nat. Rev. Neurosci. 9, 292–303 10.1038/nrn2258 18319728 PMC2631351

[B18] Falkenberg H. K. Bex P. J. (2014). Sources of motion-sensitivity loss in glaucoma. Invest. Ophthalmol. Vis. Sci. 48, 2913–2921 10.1167/iovs.06-0752 17525228

[B19] Falkenberg H. K. Simpson W. A. Dutton G. N. (2014). Development of sampling efficiency and internal noise in motion detection and discrimination in school-aged children. Vision Res. 100, 8–17 10.1016/j.visres.2014.04.001 24732568

[B20] Garcia-Quispe L. Gordon J. Zemon V. (2009). Development of contrast mechanisms in humans: a VEP study. Optom. Vis. Sci. 86, 708–716 10.1097/OPX.0b013e3181a61673 19417712 PMC2873234

[B21] Garey L. J. De Courten C. (1983). Structural development of the lateral geniculate nucleus and visual cortex in monkey and man. Behav. Brain Res. 10, 3–13 10.1016/0166-4328(83)90145-6 6639728

[B22] Geisler W. S. (2003). Ideal observer analysis in Visual Neurosciences, eds Chalupa L. M. Werner J. S. (Boston, MA: MIT Press), 825–837

[B23] Gold J. Bennett P. J. Sekuler A. B. (1999). Signal but not noise changes with perceptual learning. Nature 402, 176–178 10.1038/46027 10647007

[B24] Goldberg M. Maurer D. Lewis T. L. (2001). Developmental changes in attention: the effects of endogenous cueing and of distractors. Dev. Sci. 4, 209–219 10.1111/1467-7687.00166

[B25] Green D. M. Swets J. A. (1974). Signal Detection Theory. New York, NY: Wiley

[B26] Hecht S. Shlaer S. Pirenne M. H. (1942). Energy, quanta, and vision. J. Gen. Physiol. 25, 819–840 10.1085/jgp.25.6.819 19873316 PMC2142545

[B27] Huttenlocher P. R. De Courten C. Garey L. J. Van Der Loos H. (1982). Synaptogenesis in human visual cortex – evidence for synapse elimination during normal development. Neurosci. Lett. 33, 247–252 10.1016/0304-3940(82)90379-2 7162689

[B28] Jeon S. T. Lu Z. L. Dosher B. A. (2009). Characterizing perceptual performance at multiple discrimination precisions in external noise. J. Opt. Soc. Am. A 26, B43–B58 10.1364/JOSAA.26.000B43 19884915 PMC2829446

[B29] Jeon S. T. Maurer D. Lewis T. L. (2012). The effect of video game training on the vision of adults with bilateral deprivation amblyopia. Seeing Perceiving 25, 493–520 10.1163/18784763-00002391 23193607

[B30] Jones R. C. (1959). Quantum efficiency of human vision. J. Opt. Soc. Am. A. 49, 645–653 13655161 10.1364/josa.49.000645

[B31] Kiorpes L. Movshon J. A. (1998). Peripheral and central factors limiting the development of contrast sensitivity in Macaque monkeys. Vision Res. 38, 61–70 10.1016/S0042-6989(97)00155-7 9474376

[B32] Kiorpes L. Tang C. Hawken M. J. Movshon J. A. (2003). Ideal observer analysis of the development of spatial contrast sensitivity in macaque monkeys. J. Vis. 3, 630–641 10.1167/3.10.6 14640887

[B33] Klein S. A. Levi D. M. (2009). Stochastic model for detection of signals in noise. J. Opt. Soc. Am. A 26, B110–B126 10.1364/JOSAA.26.00B110 19884912 PMC2942087

[B34] Kontsevich L. L. Chen C. C. Tyler C. W. (2002). Separating the effects of response nonlinearity and internal noise psychophysically. Vision Res. 42, 1771–1784 10.1016/S0042-6989(02)00091-3 12127109

[B35] Legge G. E. Foley J. M. (1980). Contrast masking in human vision. J. Opt. Soc. Am. A 70, 1458–1471 7463185 10.1364/josa.70.001458

[B36] Lesmes L. A. Jeon S.-T. Lu Z.-L. Dosher B. A. (2006). Bayesian adaptive estimation of threshold versus contrast external noise functions: the quick TvC method. Vision Res. 46, 3160–3176 10.1016/j.visres.2006.04.022 16782167

[B37] Levi D. M. Klein S. A. Chen I. (2007). The response of the amblyopic visual system to noise. Vision Res. 47, 2531–2542 10.1016/j.visres.2007.06.014 17697689 PMC2099256

[B38] Levi D. M. Klein S. A. Chen I. (2008). What limits performance in the amblyopic visual system: seeing signals in noise with an amblyopic brain. J. Vis. 8, 1–23 10.1167/8.4.1 18484840

[B39] Li R. W. Levi D. M. Klein S. A. (2004). Perceptual learning improves efficiency by re-tuning the decision ‘template’ for position discrimination. Nat. Neurosci. 7, 178–183 10.1038/nn1183 14730311

[B40] Lu Z. L. Dosher B. A. (1998). External noise distinguishes attention mechanisms. Vision Res. 38, 1183–1198 10.1016/S0042-6989(97)00273-3 9666987

[B41] Lu Z. L. Dosher B. A. (1999). Characterizing human perceptual inefficiencies with equivalent internal noise. J. Opt. Soc. Am. A. 16, 764–778 10.1364/JOSAA.16.000764 10069062

[B42a] Lu Z. L. Dosher B. A. (2008). Characterizing observers using external noise and observer models: assessing internal representations with external noise. Psychol. Rev. 115, 44–82 10.1037/0033-295X.115.1.44 18211184

[B42] Lu Z. L. Jeon S. T. Dosher B. A. (2004). Temporal tuning characteristics of the perceptual template and endogenous cuing of spatial attention. Vision Res. 44, 1333–1350 10.1016/j.visres.2003.12.017 15066394

[B43] Mathworks (2008). MATLAB 7.6. Natick, MA: The Mathworks Inc

[B44] Nachmias J. Sansbury R. V. (1974). Grating contrast: discrimination may be better than detection. Vision Res. 14, 1039–1042 10.1016/0042-6989(74)90175-8 4432385

[B45] Neu B. Sireteanu R. (1997). Monocular acuity in preschool children: assessment with the Teller and Keeler acuity cards in comparison to the C-test. Strabismus 5, 185–202 10.3109/09273979709044534 21314372

[B46] Norcia A. M. Tyler C. W. (1985). Infant VEP acuity measurements: analysis of individual differences and measurement error. Electroencephalogr. Clin. Neurophysiol. 61, 359–369 10.1016/0013-4694(85)91026-0 2412787

[B47] Pardhan S. Gilchrist J. Elliott D. B. Beh G. K. (1996). A comparison of sampling efficiency and internal noise level in young and old subjects. Vision Res. 36, 1641–1648 10.1016/0042-6989(95)00214-6 8759465

[B48] Pelli D. G. (1990). The quantum efficiency of vision in Vision: Coding and Efficiency, ed Blakemore C. (Cambridge: Cambridge University Press), 3–24

[B49] Pinto J. G. A. Hornby K. R. Jones D. G. Murphy K. M. (2010). Developmental changes in GABAergic mechanisms in human visual cortex across the lifespan. Front. Cell. Neurosci. 4:16 10.3389/fncel.2010.00016 20592950 PMC2893712

[B50] Ridderinkhof K. R. Van Der Molen M. W. (1995). When global information and local information collide: a brain potential analysis of the locus of interference effects. Biol. Psychol. 41, 29–53 10.1016/0301-0511(95)05125-T 8562672

[B51] Rose A. (1948). The sensitivity performance of the human eye on an absolute scale. J. Opt. Soc. Am. A. 38, 196–208 18901781 10.1364/josa.38.000196

[B52] Shaw P. Kabani N. J. Lerch J. P. Eckstrand K. Lenroot R. Gogtay N. (2008). Neurodevelopmental trajectories of the human cerebral cortex. J. Neurosci. 28, 3586–3594 10.1523/JNEUROSCI.5309-07.2008 18385317 PMC6671079

[B53] Skoczenski A. M. Norcia A. M. (1998). Neural noise limitations on infant visual sensitivity. Nature 391, 697–700 10.1038/35630 9490413

[B54] Solomon J. A. (2002). Noise reveals visual mechanisms of detection and discrimination. J. Vis. 2, 105–120 10.1167/2.1.7 12678599

[B55] Toga A. W. Thompson P. M. Sowell E. R. (2006). Mapping brain maturation. Trends Neurosci. 29, 148–159 10.1016/j.tins.2006.01.007 16472876 PMC3113697

[B56] Yuodelis C. Hendrickson A. (1986). A qualitative and quantitative analysis of the human fovea during development. Vision Res. 26, 847–855 10.1016/0042-6989(86)90143-4 3750868

