# Supplementary material for: Developmental mechanisms underlying improved contrast thresholds for discriminations of orientation signals embedded in noise
Source: Front Psychol. 2014 Sep 8;5:977. doi: 10.3389/fpsyg.2014.00977 (PMC4157613; doi:10.3389/fpsyg.2014.00977)
Supplement: Supplementary file 1 [file DataSheet1.DOCX]

**Appendix A**. Full list of fitted *PTM* parameters for nested models. The numbers next to each model show the goodness of fit, whereas those inside the parentheses in the full model table represent the confidence intervals, calculated for the best model only.

**Full (***r^2^* = 0.9852**)**

| Age  Parameter | 5 | 7 | 9 | adult |
| --- | --- | --- | --- | --- |
| *Β* | 2.1892 (1.8276, 2.5507) | | | |
| Γ | 1.6778 (1.5144, 1.8412) | | | |
| *N_add_* | 0.0315 (0.0215, 0.0416) | | | |
| *N_mul_* | 0.3763 (0.3459, 0.4067) | | | |
| *A_a_* | 1 | 0.6139  (0.33, 0.8979) | 0.3966  (0.1418, 0.6511) | 0.3001  (0.1224, 0.4778) |
| *A_f_* | 1 | 0.5492  (0.4295, 0.6690) | 0.4229  (0.3308, 0.5150) | 0.3998  (0.3250, 0.4745) |
| *A_m_* | 1 | 0.2939  (-0.2066, 0.7934) | 0.5161  (0.0163, 1.0163) | 0.0004  (0.0004, 0.0005) |

***A_a_* & *A_f_* (***r^2^* = 0.9377**)**

| Age  Parameter | 5 | 7 | 9 | adult |
| --- | --- | --- | --- | --- |
| *β* | 1.7474 | | | |
| γ | 1.6631 | | | |
| *N_add_* | 0.0316 | | | |
| *N_mul_* | 0.2772 | | | |
| *A_a_* | 1 | 0.3827 | 0.2588 | 0.1848 |
| *A_f_* | 1 | 0.3974 | 0.3163 | 0.2852 |
| *A_m_* | 1 | | | |

***A_a_* & *A_m_* (***r^2^* = 0.9440**)**

| Age  Parameter | 5 | 7 | 9 | adult |
| --- | --- | --- | --- | --- |
| *β* | 4.4878 | | | |
| γ | 1.6251 | | | |
| *N_add_* | 0.1371 | | | |
| *N_mul_* | 0.3783 | | | |
| *A_a_* | 1 | 0.5132 | 0.3460 | 0.2433 |
| *A_f_* | 1 | | | |
| *A_m_* | 1 | 0.3803 | 0.0000 | 0.0000 |

***A_f_* & *A_m_* (***r^2^* = 0.9236**)**

| Age  Parameter | 5 | 7 | 9 | adult |
| --- | --- | --- | --- | --- |
| *β* | 2.0721 | | | |
| γ | 1.7649 | | | |
| *N_add_* | 0.0107 | | | |
| *N_mul_* | 0.3853 | | | |
| *A_a_* | 1 | | | |
| *A_f_* | 1 | 0.5037 | 0.4090 | 0.3469 |
| *A_m_* | 1 | 0.7162 | 0.2981 | 0.0016 |

***A_a_* (***r^2^* = 0.8831**)**

| Age  Parameter | 5 | 7 | 9 | adult |
| --- | --- | --- | --- | --- |
| *β* | 5.1441 | | | |
| γ | 1.4767 | | | |
| *N_add_* | 0.3308 | | | |
| *N_mul_* | 0.2024 | | | |
| *A_a_* | 1 | 0.3170 | 0.2130 | 0.1543 |
| *A_f_* | 1 | | | |
| *A_m_* |  |  |  |  |

***A_f_* (***r^2^* = 0.8226**)**

| Age  Parameter | 5 | 7 | 9 | Adult |
| --- | --- | --- | --- | --- |
| *β* | 1.2212 | | | |
| γ | 1.3249 | | | |
| *N_add_* | 0.0163 | | | |
| *N_mul_* | 0.0003 | | | |
| *A_a_* | 1 | | | |
| *A_f_* | 1 | 0.3090 | 0.2134 | 0.1692 |
| *A_m_* | 1 | | | |

***A_m_* (***r^2^* = 0.8559**)**

| Age  Parameter | 5 | 7 | 9 | adult |
| --- | --- | --- | --- | --- |
| *β* | 4.0307 | | | |
| γ | 1.8972 | | | |
| *N_add_* | 0.0284 | | | |
| *N_mul_* | 0.3890 | | | |
| *A_a_* | 1 | | | |
| *A_f_* |  |  |  |  |
| *A_m_* | 1 | 0.7436 | 0.0006 | 0.0000 |

**No change (***r^2^* = 0.5966**)**

| Age  Parameter | 5 | 7 | 9 | adult |
| --- | --- | --- | --- | --- |
| *β* | 3.9962 | | | |
| γ | 1.6583 | | | |
| *N_add_* | 0.0467 | | | |
| *N_mul_* | 0.2757 | | | |
| *A_a_* | 1 | | | |
| *A_f_* |  |  |  |  |
| *A_m_* |  |  |  |  |
