# Supplementary material for: Developmental mechanisms underlying improved contrast thresholds for discriminations of orientation signals embedded in noise
Source: Front Psychol. 2014 Sep 8;5:977. doi: 10.3389/fpsyg.2014.00977 (PMC4157613; doi:10.3389/fpsyg.2014.00977)
Supplement: Supplementary file 2 [file DataSheet2.DOCX]

**Appendix B.** Full list of statistical nested model comparison results

**Full** vs. ***A_a_* & *A_f_***: *F*(3,95) = 102.1055, *p* = 0.0000

**Full** vs**. *A_a_* & *A_m_***: *F*(3,95) = 88.4160, *p* = 0.0000

**Full** vs. ***A_f_* & *A_m_***: *F*(3,95) = 132.1948, *p* = 0.0000

**Full** vs. ***A_a_***: *F*(6,95) = 109.6069, *p* = 0.0000

**Full** vs. ***A_f_***: *F*(6,95) = 174.5229, *p* = 0.0000

**Full** vs. ***A_m_***: *F*(6,95) = 138.7348, *p* = 0.0000

**Full** vs. **No Change**: *F*(9,95) = 278.0240, *p* = 0.0000

***A_a_* & *A_f_*** vs. ***A_a_***: *F*(3,98) = 28.5974, *p* = 0.0000

***A_a_* & *A_f_*** vs. ***A_f_***: *F*(3,98) = 60.3019, *p* = 0.0000

***A_a_* & *A_f_*** vs. ***A_m_***: *F*(3,98) = 42.8233, *p* = 0.0000

***A_a_* & *A_f_*** vs. **No Change**: *F*(6,98) = 89.3718, *p* = 0.0000

***A_a_* & *A_m_*** vs. ***A_a_***: *F*(3,98) = 35.5815, *p* = 0.0000

***A_a_* & *A_m_*** vs. ***A_f_***: *F*(3,98) = 70.9004, *p* = 0.0000

***A_a_* & *A_m_*** vs. ***A_m_***: *F*(3,98) = 51.4292, *p* = 0.0000

***A_a_* & *A_m_*** vs. **No Change**: *F*(6,98) = 101.4222, *p* = 0.0000

***A_f_* & *A_m_*** vs. ***A_a_***: *F*(3,98) = 17.3477, *p* = 0.0000

***A_f_* & *A_m_*** vs. ***A_f_***: *F*(3,98) = 43.2304, *p* = 0.0000

***A_f_* & *A_m_*** vs. ***A_m_***: *F*(3,98) = 28.9613, *p* = 0.0000

***A_f_* & *A_m_*** vs. **No Change**: *F*(6,98) = 69.9615, *p* = 0.0000

***A_a_*** vs**. No Change**: *F*(3,101) = 82.5103, *p* = 0.0000

***A_f_*** vs. **No Change**: *F*(3,101) = 42.8912, *p* = 0.0000

***A_m_*** vs. **No Change**: *F*(3,101) = 60.6171, *p* = 0.0000
